# Mind wandering in reading: An embodied approach

**DOI:** 10.3389/fnhum.2023.1061437

**Published:** 2023-03-02

**Authors:** Sarah Bro Trasmundi, Juan Toro

**Affiliations:** ^1^Department of Literature, Area Studies and European Languages, University of Oslo, Oslo, Norway; ^2^Department of Language, Culture, History and Communication, University of Southern Denmark, Odense, Denmark

**Keywords:** mind wandering, embodied cognition, reading, cognitive ethnography, phenomenology, embodying mind wandering

## Abstract

In the last 20 years, the study of mind wandering has attracted the attention of a growing number of researchers from fields like psychology, philosophy, and neuroscience. Mind wandering has been characterized in multiple ways: as task-unrelated, unintentional, stimulus-independent, or unguided thought processes. Those accounts have mostly focused on the identification of neurocognitive mechanisms that enable the emergence of mind-wandering episodes. Reading is one activity in which mind wandering frequently occurs, and it is widely accepted that mind wandering is detrimental for reading flow, comprehension and the capacity to make inferences based on the text. This mind wandering scepsis in reading is based on two unchallenged views: (i) that reading is a disembodied, mental activity of information processing, and (ii) that mind wandering is essentially characterized as a task-unrelated and involuntary thought process that disrupts all kinds of goal-oriented behavior. However, recent developments within cognitive science treat the mind as embodied and thus challenge both ontological and epistemological assumptions about *what* mind wandering is, *where* it is located, and *how* it is being studied empirically during reading. In this article we integrate embodied accounts of mind wandering and reading to show how reading benefits from nested mind wandering processes. Empirically, we investigate how a reader can move successfully in and out of different embodied processes and mesh different cognitive strategies over time, including some forms of mind wandering. While such changes in reading are frequently deemed dysfunctional, we suggest an alternative interpretation: Rather than seeking constant flow and fluency, we propose that reading is multi-actional and benefits from drawing on different cognitive strategies spanning mind wandering processes and goal-oriented behavior. In that sense, we suggest that mind wandering has a potential for enriching cognitive processes underlying reading, such as imagining and reflection. We exemplify these insights through analyses of data obtained in ethnographic and semi-experimental studies of reading practices. We conclude that to capture cognitive phenomena within an embodied framework, a richer methodology must be developed. Such a methodology must not only be capable of accounting for brains, bodies, and contexts in isolation, but must consider an overall brain-body-environment system.

## 1. Introduction: Integrating mind wandering and reading

We argue that mind wandering is part and parcel of creative and imaginative reading. We further suggest that this integration often has a positive impact on reading outcome when measured in terms of imagining or creativity. Our main focus is thus not reading *or* mind wandering in isolation, but rather how mind wandering can be seen as a condition for a special form of imaginative reading. The focus thus encourages an integration of mind wandering and engaging with letters on a page as contributing to the same task of imaginative reading. We are not arguing that mind wandering by definition is valuable (or detrimental) for all cases of reading. Rather, it depends on the situation, the task and the way it is constrained, enacted and managed by the reader. Further, empirical video-observations indicate the untapped potential for linking the concepts and exploring the functional and valuable effects mind wandering can have on imaginative reading.

In the following we present the background for this integrational idea. The fact that reading is not a smooth, continuous process, but includes various kinds of ruptures and different cognitive processes (Shanahan et al., 2011; Kukkonen, 2019; Trasmundi and Cowley, 2020), encourages researchers to explore how a reader makes sense with a text, including sense making during such ruptures and breaks in which mind wandering often plays a role. The focus on mind wandering in reading is not novel. The literature on how mind wandering impacts reading comprehension and memory is rich and has grown bigger in the last decade (D’Mello, 2018; Fabri and Kukkonen, 2019; Bosch and D’Mello, 2021). Reading research has developed influential cognitive theories of the different processes [(neuro) cognitive, emotional, and experiential] that are involved in reading. However, recent studies have primarily analyzed mind wandering in relation to brain states and processes (Kucyi, 2018; Compton et al., 2019) or its experiential value (Crosswell et al., 2020) and by means of brain imaging techniques or phenomenological reports. The whole-bodied real-time processes of mind wandering in reading–that we attend to in the analysis in section “4. Methodology: A video-ethnography and phenomenology of mind wandering in reading”–are indeed under-explored. Further, while phenomenological results are valuable, they say little about the on-going processes that readers often are unaware of. In contrast to phenomenological approaches, neuroscience has shed light on what happens in the brain as readers’ minds wander (see section “2. Mind wandering in the mainstream” for a discussion). This kind of research is useful within neuroscience but provides no link to its experiential backbone. In this article, we also express a concern with the interest in universality that underlies neuroscience. Neuroscience claims to have identified the neural correlates of mind wandering processes. That is, it highlights the unified dimensions of mind wandering at a neurological level. Yet, experientially, mind wandering cannot be reduced to a universal phenomenon, hence a more complex methodology to study the conditions, emergence and constraints in mind wandering in reading across different situations, and across different scales, such as the neural, behavioral/bodily, and experiential, is needed.

**FIGURE 1:**
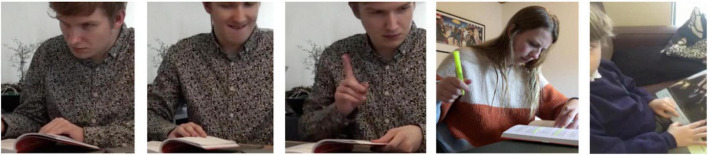
Gallery of readers’ self-initiated micro breaks in reading that are part of reading.

Thus, the assumption we will defend theoretically, that mind wandering in some cases fuels imaginative reading, stems from a recent pilot study on reading strategies. The exploratory study showed that readers continually initiate multiple micro-breaks, often leading to fast, local processes of mind wandering. The gallery below, from this explorative study, provides a few examples of such breaks in natural (i.e., non-experimental) reading situations (see Figure 1). The few qualitative examples indicate that the readers do not disengage when they elicit breaks. In fact, the breaks do not seem to relate to decline in attention. Rather, the readers use gestures (such as pointing in the text), which allow them to resume the ocular scanning efficiently.

The self-initiated micro-breaks, some of which are cases of mind wandering that often emerge in milliseconds or seconds, indeed have a particular function, yet they have not been studied systematically. We are interested in exploring this link further to understand what a different perspective on mind wandering and reading could entail for theoretical model building but also for education more generally.

Crucially, by integration we do not intend to merge the distinct processes into one of the same. We simply argue that mind wandering might not be seen as belonging to a non-task domain, and that reading involves more than continuous scanning of letters on a page. In fact, we suggest that mind wandering can make task performance in the domain of imaginative reading more elegant, creative, and original. That is, mind wandering and scanning of letters (as is our particular interest) are different cognitive processes that can be integrated to achieve the main task of imaginative reading in valuable ways. This integration, we argue, can be understood, and explored within the embodied paradigm of cognition.

In section “2. Mind wandering in the mainstream,” we describe the main challenges in integrating mind wandering and reading. We trace these challenges to historical-scientific accounts that are founded in the disembodied and cognitivist conception of the human mind, which leads to analogous conceptions of mind wandering (see Smallwood and Schooler, 2015). In this view both reading and mind wandering are treated as isolated, competing mental activities, where the latter complicates and disrupts the former. In section “2. Mind wandering in the mainstream” we further elaborate on the neurocognitive processes associated with mind wandering and we end the section by developing a proposal of how to widen the perspective to conceive those processes as strongly embodied. Likewise, in section “3. Reading in the mainstream,” we present the cognitivist assumptions underlying mainstream reading research. This model has led to the assumption that reading is a single task of decoding and interpretation, similar across contexts, thus enabled by the same set of cognitive mechanisms. We end the section by an elaboration of reading in an embodied perspective, which opens up for a new and broader take on reading as a manifold task domain. We present an interdisciplinary methodology (sections “4. Methodology: A video-ethnography and phenomenology of mind wandering in reading”) for studying the link between mind wandering and reading, which we analyze qualitatively by video-ethnographic and phenomenological methods in section “4.1. Case 1: A ruptured reading flow and the imaginative power of breaks and 4.2. Case II: A phenomenology of reading.” Finally, we discuss (section “5. Discussion: Implications and future directions”) and conclude (section “6. Conclusion”) that because there are cases where mind wandering enriches both reading experience and outcome this integration should be studied more systematically and with the intent to design and support such processes in education more generally.

## 2. Mind wandering in the mainstream

Intuitively, people know when their mind has wandered. For instance, when a person reads the news and suddenly remembers that s/he forgot to buy milk, and then starts thinking about the location of the closest grocery store. In a technical sense, the person might keep decoding the text while thinking about the milk, but attention is no longer engaged with what the person decodes. The term mind wandering symbolizes that the mind drives off-road and lays down an unpredictable path as it wanders off to task-unrelated topics. Commonly, a disengagement of the mind with the task at hand is accompanied by perceptual decoupling. This decoupling indicates that thoughts are not constrained by the events and objects in the environment, and people generate their own stream of thoughts, which is often related to pressing concerns, past events, or future plans (Randall et al., 2014; Smallwood and Schooler, 2015; Gonçalves et al., 2020). Additionally, sometimes people are aware that their attention drifts to a task-unrelated issue (tuning-out), but often they are unaware of this *in situ*, and only become aware of it after the event (zoning-out) (Schooler et al., 2004; Dixon et al., 2014).

According to an often-quoted result, between 25 and 50% of our thoughts are thematically unrelated to the situation in which we are embedded (Kane et al., 2007; Killingsworth and Gilbert, 2010). Despite being such a common feature of our daily experience, it took a very long time for researchers of the mind to study it systematically. Among the reasons for this neglect is the assumption that it was a completely hidden, mental process reserved for subjective experience. In that view, psychology’s historical distrust of introspective descriptions of experience made it an illegitimate object of scientific study (see Callard et al., 2012). However, the interest in mind wandering has been reawakened with the development of new methods to the study of the mind, including techniques of measuring brain activity like electroencephalography (EEG) and functional magnetic resonance imaging (fMRI), together with the discovery of the default mode network (DMN). The DMN is constituted by a set of brain regions centered in the medial prefrontal cortex, the medial parietal cortex, and the lateral parietal cortex (Raichle et al., 2001). A reduction of the activity in the DMN has been observed during attention-demanding tasks, such as focused reading and planning, but increase in activity during more complex and open/abstract processes, such as mind wandering, spontaneity, time-travel, and memory of abstract thinking (see Mason et al., 2007; Christoff et al., 2009). It has also been shown that dysregulation of brain areas involving the DMN can affect the person’s capacity to inhibit self-generated thoughts and attention shifting. Such dysregulation has been associated with low working memory capacity. In the same line of research, the role of emotions (both positive and negative emotions) has been shown to play an important role in the person’s capacity for action control and action inhibition (see Battaglia et al., 2022a,b). Second, the location of DMN is functionally and spatially far away from those regions associated with sensory-motor systems, which indicates that different cognitive strategies are engaged depending on the demands for cognition, for instance imagining the future vs. opening a door.

Although it is widely acknowledged that DMN plays a central role in mind wandering, recent observations have shown that the correlation between DMN and mind wandering might not be that simple. For instance, it has been observed that DMN can be flexibly coupled with other brain networks to support task-relevant cognitive functions (Elton and Gao, 2015; Sormaz et al., 2018), and that working memory performance is associated with the posterior cingulate cortex, which is a central node of DMN (Turnbull et al., 2019). These examples, among others, point to the fact that the neural mechanisms or neural signatures underlying mind wandering are not a settled matter in the scientific community (see Groot et al., 2021).

According to Klinger et al. (1973), mind wandering is caused by the high saliency of a person’s current concerns, which exceeds the saliency for the person of the ongoing task (see also Smallwood et al., 2013). McVay and Kane (2010) claim that mind wandering is the result of the person’s failure to keep executive control–the capacity to control attentional resources to accomplish tasks–against the mind’s natural tendency to follow self-generated thoughts (see McVay and Kane, 2010, 2012; Randall et al., 2014). Unlike McVay and Kane’s proposal, Smallwood and Schooler (2006), (see also Smallwood et al., 2012) claim that mind wandering is *not* a failure of executive control on attention, but rather an independent process competing for attention against the task-related stream of thought. Leaving aside the discussion about which theory explains best the cognitive mechanisms underlying mind wandering, let us emphasize that they all operate within the cognitivist paradigm, which conceives the mind as an information processing machine. By and large, theories of mind wandering have implicitly adopted the bottleneck conception of attention developed by Broadbent in the 1950s (see Broadbent, 1958; Watzl, 2017).

### 2.1. Mind wandering, task, and function

Naturally, a fluctuation of attention in which the mind wanders to task-unrelated thoughts like future plans, past events, or pressing concerns, has an impact on the task being performed if such task has not been automated by the person. That is also the reason why mind wandering has been reported to compromise reading comprehension (Schooler et al., 2004) as well as the ability to create a situation model—through which the reader establishes connections between events in the text and general knowledge, and finally makes inferences based on those connections (Smallwood et al., 2008). However, the results of these studies have important nuances and caveats that are worth considering. For instance, Smallwood et al.’s (2008) study shows that there were significant differences in the impairment of participants to generate a situation model, depending on whether they were aware of their mind wandering or not (tuning out vs. zoning out): “when mind wandering was separated by awareness, reports of zoning out were a reliable negative predictor of comprehension (…) whereas tuning out was not” (1,149). This means that not all forms of mind wandering are necessarily negative on task performance, such as reading comprehension for instance. Also, in Smallwood et al.’s study, the conditions in which participants engaged with a text reflect an implicit conception of reading. In this study, the text was presented in black on a white screen “word by word, which denied participants the opportunity for regressions to previous parts of the text” (1,149). Surely, there are methodological and practical reasons supporting this design, but it is important to make explicit that the kind of reading being studied is a very specific one, namely, linear, progressive reading. This form of reading is part of the cognitivist conception of reading, which also includes other characteristics, like fluency, accuracy, and speed (see Toro and Trasmundi, 2023). Below we introduce an embodied alternative to this brain-bound view on mind wandering.

### 2.2. Switching paradigm: Embodying mind wandering

Up to this point we have proposed that mind wandering is a heterogeneous phenomenon that has diverse effects on reading. We have also emphasized that our current understanding of mind wandering and of reading is grounded in cognitivist assumptions about the mind, which necessarily permeate the investigations on the impact of mind wandering in reading. We propose to re-conceive reading and mind wandering within an embodied framework. In an embodied conception of reading and mind wandering, the relation between those phenomena should not necessarily be as troublesome and detrimental as previously assumed. In fact, we suggest that mind-wandering is linked to many cognitive processes in crucial and beneficial ways, as it provides both motivation and potential for engaging in tasks more creatively, originally and critically. Below we introduce the embodied cognition paradigm and apply it to mind wandering.

The paradigm of embodied cognition suggests that neurological, bodily, and environmental processes are constitutively involved and densely intertwined in all kinds of cognitive processes (Gallagher, 2006; Steffensen, 2013; Varela et al., 2016; Loaiza et al., 2020; Trasmundi, 2020). In line with the enactive approach, it conceives cognition as an embodied skill in which meaning emerges from sensorimotor patterns of perception and action (Thompson, 2007; Di Paolo et al., 2018). That is, the brain does not process information in computationalist terms, rather, meaning is created through the coupling of brain, body and environment (see Thompson, 2007). By characterizing cognition in this way, the framework encourages integration of data and conceptual analyses from very diverse sources and disciplines, including neuroscience, anthropology, ethnography, philosophy, and psychology. Although the very role that embodiment plays in cognition is a matter of dispute within embodied cognitive science, we endorse what has been termed *strong embodiment*, which refers to “any view that gives a clear explanatory role to the body,” as opposed to weak embodiment, for which the explanatory role falls on the representations of the body (Alsmith and de Vignemont, 2012: 3).

Along with the increased interest in mind wandering, a myriad of characterizations of the phenomenon was developed, to a point in which it became hard to know what mind wandering essentially amounted to. Is it essentially task-unrelated thought (Smallwood and Schooler, 2006), or unintentional thought (McVay and Kane, 2010; Bixler and D’Mello, 2016), stimulus independent thought (Smallwood and Schooler, 2015), or unguided thought (Irving, 2016)? Seli et al. (2018a,b) conceive the relation among the wide diversity of thought processes—currently labeled in the literature as mind wandering– as that of a *family resemblance*, analogous to Wittgenstein’s (1953) famous argument on how to define a *game*.^1^ An alternative position rejects the family resemblance approach and argues instead that mind wandering is a scientific *category.* Mind wandering, as a category, has an essential feature, that is a specific kind of spontaneous thought that emerges under weak constraints. This conception of mind wandering, originally developed by Christoff et al. (2016), has gained considerable popularity within the neuroscientific community (see, for instance, Andrews-Hanna et al., 2018; Kucyi, 2018; O’Callaghan et al., 2021). Further engagement in this debate would lead us astray, yet thinking of mind wandering in terms of spontaneous thought processes and weak constraints has important benefits in relation to our current aim: (i) it can be applied to reading,^2^ (ii) it is compatible with the embodied cognition framework, and (iii) it contributes to understanding mind wandering in relation to other crucial processes in reading, such as creative thinking and goal directed cognition.

Christoff et al. (2016) treat different kinds of thought processes in relation to the constraints that act on their emergence and dynamics at any moment. Some of those constraints are deliberate, and the agent exerts them through executive control, for instance when a person attends a boring lecture and forces herself to maintain focus on the topic (see Kane et al., 2007). Yet, other constraints are automatic, such as affective or perceptual saliencies that redirect the stream of thought independently of conscious intention. An example is when a fly starts buzzing in the room and the attention is immediately directed toward it. According to Christoff et al.’s model, goal-directed thoughts are characterized by strong deliberate constraints, that is, the agent exerts considerable executive control over the cognitive processes. In turn, cognitive processes that run more freely (i.e., subjected to weaker constraints) are spontaneous thoughts. There are three sorts of spontaneous thoughts: (i) dreaming, on which the weakest constraints are exerted; (ii) mind wandering, with relatively weak constraints; and (iii) creative thinking, with slightly stronger constraints (see Christoff et al., 2016).

At the neurocognitive level of explanation, Christoff et al. (2016) observe a link between weakened constraints on thought processes and the activation of a specific region of the DMN located on the medial temporal lobe, DN_mtl_ as Christoff et al. (2016) call it. An indication of the link between DN_mtl_ and weak deliberate constraints on thought is that in cases of zoning out, which correspond to the weakest constraints in mind wandering, DN_mtl_ shows stronger activity than in tuning out, in which constraints are stronger. Christoff et al. (2016) also call attention to the relation between DN_mtl_ and several other important cognitive processes, including spontaneous memories and spontaneous mental simulations (Gelbard-Sagiv et al., 2008). Further, DN_mtl_ is active right before the arising of spontaneous thoughts (Ellamil et al., 2016), and the hippocampus—a central part of the medial temporal lobe—is involved in episodic memory (Stark and Clark, 2004; Moscovitch et al., 2016), as well as in imagination of novel scenarios and situations (Buckner and Carroll, 2007; Hassabis et al., 2007; Buckner et al., 2008; Buckner, 2010), and in constructing new spatial scenes (Hassabis and Maguire, 2009) and finally in imagining potential future experiences (Schacter et al., 2012). Despite the clear relevance of these discoveries about the processes in which the DN_mtl_ is involved, the unit of analysis is exclusively the neurocognitive process(es). From an embodied perspective, we defend that processes, like imagining new scenarios or simulating a situation, build on crucial neurocognitive components, but are not limited to them: bodily and environmental elements are constitutive of such processes too.

Ilundáin-Agurruza’s notion of *corporeal imagining* (CI) is a good example of what we mean by imagination being embodied. He defines CI as those imaginative processes that, unlike eidetic imaginings, “originate in and are expressed by our corporeal involvements with the environment” (2017: 97). To apply this notion to the simulation of a situation, Ilundáin-Agarruza discusses the case of Alex Honnold, a rock climber who is about to solo climb for the first time near a vertical 360-m sandstone wall. Before climbing, he spends 2 days “sitting and thinking, hour after hour. Visualising every single move, everything that could possibly happen” (Honnold and Roberts, 2016; Ilundáin-Agurruza, 2017). Honnold’s imagining process is a case of CI, since it is not only pictorial, but it involves kinetic, tactile, kinesthetic, nociceptive, and other sensory modalities that are deeply embodied in nature (see Ilundáin-Agurruza, 2017). Furthermore, eidetic imagining is, according to Ilundáin-Agarruza, dependent on corporeal imagining (see also Dewey, 1980).

However, this analysis not only applies to cases where embodiment is explicit in imagination. Even when the person imagines something that seems to be entirely independent of their embodiment, their body is involved in crucial constitutive ways. Let us suppose that a person follows Rucińska and Gallagher (2021) in imagining a snowy mountain top. They imagine it in terms of their movement possibilities and the accompanying kinesthetic sensations, they imagine it from an egocentric perspective and from the position that their body would occupy, and their imagination is also affectively loaded: if the person is in a good mood and filled with energy, the mountain appears as climbable, whereas if the person is tired, it will appear less climbable (see Gallagher and Bower, 2014; Rucińska and Gallagher, 2021). Now, since mind wandering is a close relative of imagination, creative thinking, dreaming, and analogous processes, there are strong reasons to think that an embodied account of it can be developed.

Our proposal to embody mind wandering is based on enriching how we conceive the lowering of deliberate constraints on thought processes hypothesized by Christoff et al. (2016). We propose to conceptualize the lowering of deliberate constraints on thought processes, partially enabled at the neurocognitive level by the activation of the DN_mtl_, as coupled with embodied processes enacted by the cognitive agent. The main concern is, then, how the modulation of lived temporality by the cognitive agent contributes to the lowering of constraints on thought and to the emergence of spontaneous thoughts. Let us recall that in Christoff and colleagues’ dynamic model from weaker to stronger deliberate constraints, we find the following thought processes: dreaming, mind wandering, creative thinking, and goal directed thought, respectively. These categories constitute a spectrum in which borders are fuzzy, and deliberate constraints vary continuously from weak to strong, depending on the dynamics of the activity taking place. Importantly, modulating the lived temporality of those cognitive processes allows for a variation in the strength of deliberate constraints. Think of a word you read that suddenly sounds strange and makes you engage with the sound—or aspects of the sound—only for a millisecond before resuming to the reading. Creative thinking and mind wandering demand a diminished cognitive control on the stream of thoughts, thus opening up the space for unorthodox associations of ideas, or alternative perspectives and feelings on the tasks at hand. Modulating the cognitive control on the thought processes is densely coupled with embodied processes of self-initiated ruptures or modulation of the temporality at which the task is unfolding.

Finally, agents permanently modulate the control over their cognitive processes by means of embodied strategies: a person changes her bodily posture to adopt a more focused attitude and increases the constraints on her thought processes. This same person might notice that a noise coming from outside is distracting her, so she closes the window to reduce automatic constraints. At some point she can feel compelled to let her thoughts run more freely, so she looks through the window for a moment, or closes her eyes, or stands up. In that sense, the person’s attempt to decouple from the here-and-now is indeed constrained by embodied embeddedness. The person flexibly adapts to both goal-directed thought processes and mind wandering in the situation, and the boundaries between them are fluid. In this view it makes sense to understand the processes as emerging on a cognitive continuum, rather than thinking in terms of task-switch. Mind wandering *might* be good for reaching the goal in a creative and motivated way but without following rules and analysis or predicting outcome—hence that is the point. Again, increasing or lowering the cognitive control over thoughts and modifying the environment in ways in which seems fit to the demands of the activity at hand are embodied processes that encompass the nervous system, the body, and the environment in which a person is embedded. That environment might include other persons, and social norms that guide behavior in different contexts. In the analytical section “4.1. Case 1: A ruptured reading flow and the imaginative power of breaks and 4.2. Case II: A phenomenology of reading,” we discuss how such embodied modulations of constraints over thought processes can be identified in reading.

## 3. Reading in the mainstream

Reading models are not invented in a scientific vacuum. Therefore, the discussion of reading is interdependent with how people engage with symbolic material at a given historical time. Historical processes have shaped the axiomatic and scientific bases of reading models (Wolf, 2008; Manguel, 2014).

With advances in experimental psychology and neuroscientific methods to study the brain, the reading research, according to Stanislas Dehaene, recently transformed into “*a true science of reading”* (Dehaene, 2009: 1). Neuroscience emphasizes the mentalistic dimension of reading by reference to brain mechanisms that allow the reader to make letter-sound correspondences. In such a view, there is no need to be concerned with changing ecologies, in which reading is conducted, because the underlying brain mechanisms are fixed, even universal, and thus predict effects across tasks and contexts (Dehaene, 2009). Reading is fundamentally the same across all kinds of situations. In short, explaining reading simply by reference to a set of basic mechanisms is reductionist and does not fit either empirical evidence or historical accounts of how reading has changed and continues to change (Saenger, 1999; Trasmundi and Cowley, 2020; Ingold, 2022; Trasmundi et al., 2022).

At its core, reading research is fundamentally interdisciplinary (Mangen and van der Weel, 2016; Moje et al., 2020). A recent review on reading models (Davoudi and Moghadam, 2015) also documents that extant literature on reading is unevenly developed and the number of models is unhelpfully high (Trasmundi and Cobley, 2021). Regardless of the different epistemologies underlying those models, they all share a similar focus on interpreting information to uncover or create linguistic meaning in or from the text. The view comprises 5 components in reading, which are commonly agreed on in the field: (1) *phonemic awareness*, (2) *phonics/phonetics*, (3) *fluency/speed* (4) *vocabulary*, and (5) *comprehension* (Mehta et al., 2005).

While the five components resurface in mainstream models in various ways, they ascribe explanatory power to different units in reading such as the brain (Dehaene, 2009), the hands and body (Kiefer and Trumpp, 2012; Kukkonen, 2016, 2019; Di Paolo et al., 2018), and the environment (Foley, 2012). Regardless of the explanatory basis for reading, such models are concerned with how texts relate to symbolic meaning and conceptual comprehension, and they are uninterested in the processes that are *not* causally dependent on symbolic meaning in the text, for instance when a word sounds peculiar and prompts the reader to break the reading flow and “taste” the sound and feeling of the word (Trasmundi and Cowley, 2020).

Further, in reading, the topic or “object” is classically that of using language or, in a popular formulation, making sense of written signs. One reason for this exclusive focus might be that the study of object has been defined as the text-reader relation, and breaks (including mind wandering) are assumed to be cases of task switching: they are not part of reading. A mentalistic framework can, of course, partly explain how reading deficits, such as dyslexia, can be approached in successful ways by reference to how neural circuits are constructed. The critique is thus *not* that the brain is not a central organ in reading or that reading is not about symbolic understanding. Rather, while neural functions such as making letter-sound correspondences say much about statistical knowledge of how to combine letter to sound, they say little about meaning-making and imagining, which other fields, such as literary research, social anthropology or phenomenology can shed light on. A neural, and fixed task-based explanation is insufficient in itself to explain meaning, and it is biased by Western, naïve, and cognitivists understanding of what reading involves (Ingold, 2022). How one reads depends on the overall reading ecology; for instance, if you read right to left or left to right, or from top to bottom, and whether you engage with old, printed tomes or iPads and Kindles (Ackerman and Goldsmith, 2011; Schilhab et al., 2018). In that view, it seems insufficient to treat reading as a single kind of phenomenon, and in taking the Western reading practice as the default image of reading. This image involves sitting down with a text and silently scanning the pages with the eyes, yet it is in fact historically the exception (Ingold, 2022). Furthermore, silent reading seems to be more of an ideal than an empirical reality as we will show in the analytical section. In this article we zoom in on how university students engage in mind wandering in academic reading. However, because reading is not one thing, we encourage researchers to consider mind wandering in relation to an array of constraints on the reading processes, including reading culture, personality, genre, task, text length, and the materiality and substrate of the medium; or in other words paying attention to how mind wandering functions in various reading ecologies.

Before we go further into the analysis, we present an emerging embodied perspective on reading, which broadens the view of what reading is and by doing so also welcomes processes that are not traditionally considered part of reading, such as mind wandering.

### 3.1. Reading is embodied and not silent

In the embodied perspective, reading is something a reader *does* as s/he engages with the socio-material world. Importantly, the bodily engagement changes during reading and correlates with the operation of different cognitive strategies. By investigating an array of factors including neuronal processes, embodiments, and the reading context we treat all those factors as constituents of a coherent cognitive system that enables reading. As mentioned above, the idea is that brain processes are interactively dependent on body-world engagement, hence different cognitive processes both emerge from and shape such systemic changes.

An embodied account of cognition thus requires a systemic description of how brains, bodies and environment co-function as one system. Recent neuro-philosophical accounts of human agency suggest that the brain is interactively related to the encultured body. Anderson (2014: 161) underlines: “The brain is a dynamic information-processing system that responds to and transforms structured signals from the environment in the service of generating adaptive behaviour. Sensory inputs induce patterns of activity in the brain that depend on both the nature of the input and the dynamic state of the brain. Those patterns interact in various ways.” In going beyond phrenological and modular explanations of the brain’s function as fixed, Anderson (2014: 7) suggests that “neural structures originally evolved or developed for one purpose will be reused in later emerging functionality.” So, because our brain is not originally designed for reading, more basic mechanisms must be reused and adapted for that activity. That means that any cultural artifact can allow new neuronal structures to emerge. Yet, the plasticity of the brain is not unconstrained: A cultural artifact—such as the alphabet—must find its neuronal niche where the already existing neuronal circuits are close enough to the required function and plastic enough to be recycled (Dehaene, 2009). The idea of the interactive brain binds brain, body, and world as one cognitive system just as it binds multiple timescales and pace such as past and present or fast and slow.

While embodied approaches to reading have been heavily invested in studies that bring forth valuable understandings of how media and materiality afford different tactile, embodied engagement which impact understanding (Delgado et al., 2018), their task-based epistemology of reading remains solely on how symbols trigger meaning. The epistemological belief in reading as a task of textual/symbolic understanding, ignores the ruptured, messy nature of reading and deems breaks as “outside” the task; as a decoupling, or simply a task switch. The challenge with a text-coupled task view is that actions that do not appear to be directly related to the text-scanning, are, in psychological terms, dysfunctional for task management. In reading, such a task-based epistemology carves out the unit of analysis as “continuous textual interpretation” enabled by mechanisms that fit whatever theoretical framework is applied: visual perception, embodied control of attention etc. As a result, educational systems train reading skills such as reading speed or fluency as means to achieve best reading practices. Of course, this focus leaves aside the function of breaks, or at worst teachers train students to avoid them. The argument underlying this section is that, although embodied approaches to reading have changed our understanding of the sense-making apparatus from brains to bodies (and even historical persons), reading researchers have not changed the underlying epistemology of reading.

However, from recent empirical studies on reading, the ruptured nature of reading is brought to the fore (Kukkonen, 2019; Trasmundi et al., 2021). Those studies apply an embodied approach to explore more open-endedly how reading is managed in natural settings. From those studies it becomes evident that a reading epistemology –that is capable of including symbolic interpretation, but also processes that appear to play a significant role beyond this ocular scanning and interpretation task– must be developed. Such an epistemology considers an array of activities that go on as readers engage with written material. For instance, every rapid embodied adjustment and gesture the readers’ bodies make; how they speed up or slow down; their eyes’ rapid saccading, and how they look up and away from the page, how they impose rhythmicality, stop, continue, go back, make connections and free associations, how they leaf through passages, point to the material, put it down or closer to them, and generally how they experience emotional responses which consist of much more than the “linguistic meaning” of the words they read. Readers constantly make embodied-affective judgments (Trasmundi and Cowley, 2020; Macrine and Fugate, 2022; Trasmundi et al., 2022). That is, reading involves multiple breaks. Yet, the function of those breaks is not systematically described in the reading literature. We suggest they are closely related to mind wandering. An embodied approach to reading thus allows for a broader perspective on reading which includes processes beyond the fixed single task-based understanding. As such, it is in this embodied understanding of reading that mind wandering becomes a central link, because it changes the focus from a fixed, well-defined task to a manifold task domain, in which many processes become valuable potentials, depending on which specific task (in the context) one seeks to achieve. In imaginative, creative reading, we suggest that mind wandering, both as tuning out and as zoning out, is a valuable catalyst.

The historian and literate Christian Benne emphasizes the messy features, such as imagining and breaks, in the study of reading. In an analysis of Chapter VIII of St. Augustine’s *Confessions*, he shows how what appears to be a case of silent reading, has in fact never been silent. His claim is a paradox as the chapter serves as one of the very first mentions of silent reading, and thus re-enacts an understanding of reading as a paradigm case of the operations of the Cartesian mind, where mental decoding of linguistic meaning constitutes the practice of reading. However, Benne’s analysis reveals the exact opposite:

“The scene of reading depicted here is couched in tearful, emotional upheaval. The “silence” with which he reads mainly serves as a contrast to the turmoil of the rest of the scene. It is *itself* a gesture, namely a gesture of absolute concentration and immersion in the word of God that ultimately inspired the conversion. It is certainly not a neutral description of a default technique of interpreting writing. What is more, the narrator’s silent reading is only a short parenthesis in a much larger scene that starts well before it. It begins with a strong affective expression, a pathos formula of despair, where he casts himself down. When he hears the voice that tells him to take (up) the book and read (“tolle, lege”), he immediately alters his body position again before he follows the instruction. The gesture of taking the book—which for Augustinus would already have meant a codex rather than a scroll—is by no means exterior to the act of reading, but a specific method of it.” (Benne, 2021).

By demonstrating that reading is something more than cognitive processing of linguistic meaning, Benne’s emphasis on its gestural, tactile and dialogical aspects gives a direction for how empirical work should always consider the *scene* in which the reading is performed, but also its historical dimensions (i.e., how one reads one’s world). He elaborates how reading in this case “involves a radical repositioning of the body in a vertical direction (casting down, rising, sitting down), weeping, grabbing, pointing, opening and shutting of the book, leafing through pages.” (Benne, 2021). While such embodied shifts in attention—and in particular breaks from the scanning of words–are crucial for the way meaning emerges and sediments, the conditions, emergence and function of breaks have not been investigated empirically. It is this gap we explore further in this article and suggest that the link to mind wandering will be useful.

Generally, the embodied perspective provides an alternative model that also considers how actual embodiment impacts reading. While movement does not amount to comprehension, we suggest that breaks from scanning create temporal-cognitive loopholes [that is a break from the scanning in which the reader can explore ideas (automatically or intentionally)], which allow more abstract forms of thinking to emerge. Breaks are potentially crucial for mind wandering, imagining and other forms of linking what is being read to a broader context or life history.

From an embodied perspective reading concerns how the reader (brain-body) and environment co-function as one system, and how the reader—as part of the system—uses lived temporality to modulate attention in the socio-material setting (Kukkonen, 2016). For reading research the embodied turn therefore entails an empirical *reorientation* where different cognitive strategies can be observed empirically to a larger extent than previously assumed. An embodied perspective on reading thus takes an epistemological leap forward in shaping the research in the field. No current models can explain how creative and imaginative power of reading emerges, manifests itself, and leads to enhanced skills in perspective-taking, emotional sensitivity, and nuanced cognitive judgments *beyond* linguistic, symbolic understanding. The reason for this inability, we claim, is that researchers have assumed that it is the brain that reads in predictable ways. Instead, the embodied and distributed approach to reading rethinks the localization of reading and turns to its embodied and actional dynamics. Because cognitive strategies are embodied, they must often reveal themselves as changes in the overall reading trajectory.

## 4. Methodology: A video-ethnography and phenomenology of mind wandering in reading

Our argument, that mind wandering can be conducive for certain kinds of imaginative and creative reading processes, is explored qualitatively, and from an embodied perspective by turning to actual cases of how readers engage with texts. Overall, we present data from a video-ethnographic study and a semi-experimental study that both explore how university students read different kinds of texts.

Cognitive ethnography is defined as the methodological framework which encourages temporally and spatially extended observations of a given phenomenon in its natural habitat (Hutchins, 1995; Trasmundi, 2020). It deals with cognitive aspects of human behavior, and it emphasizes embodied approaches to the study of cognition. In the studies we have explored in great details what *happens* in various settings (spatial extension). Further, we explored natural reading conditions by asking *why* breaks emerge and what happened in those breaks, and applied embodied cognition theory to examine how different constraints (cultural, personal, and socio-material) impact reading processes in both micro and macro timescales (temporal extension). The framework is an interdisciplinary methodology. The article’s aim cannot be achieved by applying only one approach or method within interaction studies, linguistics, cognitive (neuro) science, or ethnography. Likewise, neither qualitative nor quantitative methods, in themselves, will suffice. While we have used cognitive ethnography as the overall frame in which data from university students’ natural reading practices are conducted, it also includes semi-experimental studies, where we asked students to read a text in an office at the university when they were being video-recorded. The students had no possibility for choosing the location, the setting, the media or text. Several phenomenological interviews were conducted as we were particularly interested in exploring how perception of reading and observation of reading processes correlated or diverged.

The examples we present here do *not* serve to generate explanatory power of our idea (e.g., in terms of identifying law-like mechanisms for how the integration of mind wandering in reading is enabled; neither is the aim to argue for the reproducibility of the cases). Instead, and in line with cognitive ethnography (Hutchins, 1995), the aim is to illustrate real-life *particular* cases of how imaginative reading *can* and in this case *does* draw on multiple cognitive processes, such as symbolic interpretation and mind wandering. As such, much reading does not involve mind wandering, and in some cases mind wandering is indeed detrimental for task maintenance, yet in some cases it can be valuable for reading. This insight calls for further research on (a) the features that seem to scaffold/afford such positive effects, and (b) the larger circumstances that impact how it is enabled, managed and (c) whether it can be applied in strategically intelligent ways. The data here are selected to emphasize such particular instances of mind wandering in reading that have rarely been studied before. Our methodology thus opens up new questions to be addressed about the nature of mind wandering and reading as well as the link between them from various disciplinary starting points. This explorative analysis is a first step toward a more systematic investigation of those phenomena.

The analysis below falls in two parts. First, section ““4.1. Case 1: A ruptured reading flow and the imaginative power of breaks,” an ethnographic analysis of a person that recorded his reading sessions over a period of 6 months. Second, section “4.2. Case II: A phenomenology of reading,” a phenomenological interpretation of an interview in which a participant in another setup describes her experience of reading an academic text. Together the analytical parts show how cognitive processes (e.g., scanning and wandering) are constrained by different embodied strategies. Together, the qualitative excerpts emphasize cases of how cognitive processes are conditioned, how they emerge, and how they impact the overall reading task. The aim is to show how, in different contexts, readers engage with texts and use embodied strategies to control their cognitive-emotional processes during reading. Again, the cases are not intended to demonstrate a hypothesis. Rather, we aim here at strengthening the general intuition that the embodied approach constitutes a fruitful framework to understand processes like mind wandering in reading. For this reason, we abstain from presenting an exhaustive and rigorous description of the methodologies employed and invite the reader to see for themselves in daily life what we make explicit here. It is in this Wittgensteinian spirit of showing rather than saying that we discuss the following two cases.

### 4.1. Case 1: A ruptured reading flow and the imaginative power of breaks

**FIGURE 2:**
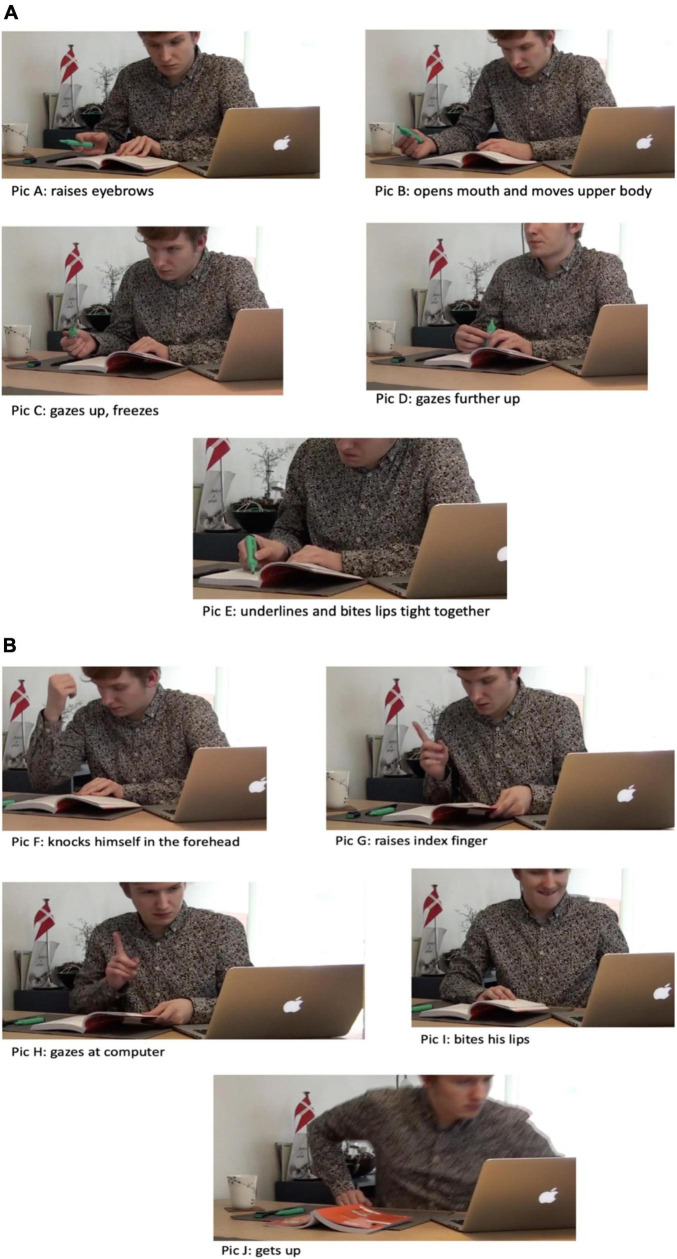
**(A,B)** Breaks from visual perception of symbols in the text.

Our first case centers around a third year, male university student who reads an academic text at home. The academic text is part of the curriculum and deals with communication and culture studies. The reading task is to comprehend and remember core concepts. The alleged outcome of this reading is that, by knowing the concepts, the student will be able to engage in critical discussions in class the following week. The student reads at home by the dining table. He has organized his workspace prior to the reading where his printed textbook, computer, and highlighter are located in front of him, just as he has made himself a cup of coffee. From the very onset the student’s reading is characterized by a dynamic, ruptured reading flow. The gallery below will be used to illustrate this in detail (see Figure 2).

He constantly hesitates, gazes up, down and around, he underlines, and his changing mimicking is significant: he raises his eyebrows (pic A), smacks with his mouth (pic E), and his visual attention shifts between ocular scanning and fixation in and beyond the text. An ethnography of his reading reveals how messy, ruptured and embodied-affective his reading generally is. The gallery above and below provides a snippet of his reading behavior. Pictures A-E reveal the first couples of minutes of his preparatory reading.

When, in reading, something triggers the reader’s experience, the affective-embodied engagement changes significantly as we see above. We have no intention of claiming *what* the reader experiences, or how he thinks and feels; rather, we point to the tensions that constantly emerge during his reading. As we argued above, tensions are exactly what prompt and constrain readers to adapt, judge and change the course of events. For instance, we treat the reader’s observable emotional reactions and cognitive agency as a sign of critical reflection which causes some sort of cognitive dissonance between the reader’s understanding and the construction of understanding the text. The reader, we argue, relies on a phenomenology of reading which prompts judgments and responses in accordance with the reader’s identity and context. We turn to how such promptings are visible in readers’ embodied material engagement, and not as traditionally approached as a result of mental, silent, and internal operations. For instance, our reader suddenly reaches an impasse, and the way out of this fixation is observed in embodied attention shifts. The analytical principle underlying such ethnographic description is identification of affective-embodied changes invoked by the reader himself (in contrast to automatic constraints such as external interruptions).

The reader manipulates the situation by using embodied strategies that make the emergent tensions manageable. Just before this sequence, the student reads rather fluently. Suddenly, he reaches an impasse and literally stops scanning (the symbolic interpretation process is put on hold). Instead, he knocks himself in the forehead three times with his right fist as he enacts a rhythmic back and forth movement with his upper body (Pic F). In our conceptual embodied framework, the reader’s mind wanders for a few seconds (obviously he does not read). While we cannot explain what he experiences, we can observe what happens in those moments, and the effects are clear: the self-initiated break prompts a row of consecutive actions. He points with his right index finger and opens his mouth but without saying anything (Pic G). Those embodied actions are performed at a fast pace and indicate a change from the almost fixated impasse to the rapid pointing. The pointing is interesting in a number of ways. First, it indicates a strategy for constraining cognitive tensions. By making the tension empirically visible in gesture, he carves out a moment in space that requires his conscious attention. The social anthropologist Tim Ingold elaborates on such tensions emerging from being “hold back” and wanting to transgress the impasse in which one is physically embedded. He describes this tension in relation to an imaginative process:

That is always shooting of in the distance; and a material engagement that is always holding us back. And this is a particular tension that humans experience. Any artist will agree on that (…). Imagine you are a composer, and the music is shooting ahead in your imagination, and you are struggling with this pen and paper trying to notate it down on manuscript paper. And a really hard work of composition is holding it there—the imagination—so you can get it down. And there is a constant anxiety that it will all going to slip away from you before you manage to catch it. And I think that is really the root of human life—the imagination (Ingold, 2021).^3^

We argue that this tension is extended for some moments. There is something he needs, but the reader is unsure what and where it is as his embodied actions reveal: After he has raised his index finger, he starts searching for something. He turns the pages quickly back and forth and he picks up the pen but leaves it soon again. He stops and gazes at one page while moving his lips repeatedly to the left and right and nods slightly. He then gazes at the computer screen for a couple of seconds before he resumes the page turning. Suddenly as he reaches the end of the chapter and perceives a new headline, he once again raises his right index finger (Pic H) and gazes toward the screen and starts doing all sorts of different actions: again, his mimicking is significant (Pic I), he gazes interchangeably toward the screen and the pages, reaches for the pen and puts it down again. Then he turns the book around and leaves to make a cup of tea (Pic J). The puzzling has been going on for approximately 2 min before he leaves the reading situation for a moment (Pic F-J).

Obviously, the student was affected by the perceptions and acted in various ways to grasp and tame those perceptions. Again, while we have no insight into “what” the student is thinking, we can easily point to the inter-bodily dynamics of his cognitive work (the how). His use of embodied strategies to manage the tension involved a distancing (he gazes up, freezes) and detours (page-turning and looking/searching at the computer screen). Something struck the reader, and he immediately modulated his attention toward certain points in the book and screen that altogether would allow him to move on. To sum up: he uses his index finger to make a spatio-temporal mark that somehow becomes an empirical cue of what he is doing and how he anticipates a need for ordering a row of cognitive processes. That is, his actions involve a detour that displaces him in relation to the reading and the actions thus allow him to keep track of what he is thinking and what he is investigating. The finger becomes a distributed component in his cognitive work (Hutchins, 1995) that allows him to hold on to a thought while investigating something related. The bodily movement becomes associated with a thought in time, and when repeated later, reminding him thereof. Eventually, he seems to be at a point where he is close to having constructed an understanding: he seems satisfied and nods as he realizes the new chapter’s headline.

The excerpts are rather representative for the overall study of readers’ engagement with preparatory texts. Reading trajectories are messy, ruptured, and dynamic, and the strategy used can be described as modulating attention enabled by using simplex tricks (pointing, delaying, displacing, and freezing) which eventually allows the reader to change the reading pace from fast to slow and vice versa. It allows the reader to infuse slow thinking with task-management. We suggest that changes in his pacemaking during reading can be seen as a continuum of thought processes spanning moments of mind wandering and cognitive task engagement. As such, mind wandering seems to be of value for his affective-cognitive engagement with the reading. We relate this kind of observational study with phenomenological reports of students’ experiences of various thought processes during reading.

### 4.2. Case II: A phenomenology of reading

The notion of pacemaking and Christoff et al.’s dynamic framework allow us to interpret the experiential reports given by the reading participants in another, semi-experimental study. In this study, participants were asked to read an academic text and a short literary story in an office at a Scandinavian university. Like in the other study, the students’ readings were video recorded. After their readings we conducted a phenomenological interview with each of the participants. In this section, we focus on a reader, M, who gives a rich description of how she experienced reading the academic text. Her experience was characterized by frustration and interruption, and she utters: *“. I sat and read the same thing, like this, five times in a row, you know, I completely lost, you know what to say, focus*.” Her description corresponds to a specific kind of mind wandering, namely, zoning out (see Smallwood and Schooler, 2015). Specifically, she loses focus and starts thinking of something non-task related, even though she tries not to, and she is being caught in a fixation loop. Her experience is that this “just happens” and she is unaware, in the moment, of what enables her thoughts to drift away. Further, she is aware that she is reading *something*, but she is incapable of making sense of *what* she is reading. It is, as she says, an “interrupted” experience: “*I sit and read the same sentence almost five times, without really knowing what it is I am reading, but I am reading something. I might jump a little more between the words, but I read and so, you know, I did not understand it all, and then I go back, so it becomes very much like what to say, back and forth, back and forth, and you know, yes, a ruptured feeling, you know.*” While M is trying to make sense of what she is reading, she experiences the need to move her body: “*I can feel that I start to sit and tilt my feet, so that my body has to move*.” The need to move reveals a bodily strategy emerging from the tension she describes. She mentions how frustration emerges as she reads and how she tries to manage this tension through a different kind of embodied pacemaking. In the interview, she elaborates: “*had it been at home, I might have left to drink a glass of water*.” She feels the urge to distance herself physically from the text because she feels trapped and unable to get into flow and to make sense of the text in the current context: “*sometimes all the words there, you know, I just think, you know, my flow is not there, so, you know, I just lose completely. and then I lose interest, that is, in the reading*.”

What is it about the setting that she feels is inhibiting her from making sense of the text? She emphasizes that reading with the window behind her makes her feel uncomfortable: “*again*, *had it been at home, I would never have sat with my back towards the windows. I would always have faced the windows*.” And she adds, “*if it had been at home, I would have thought about all this, then I would have sat, you know, then I would have sat and gazed outside, (*…*), then I would, most likely, have sat inside my living room at the dining table and, like gazed outside*.” M’s description of the optimal, natural reading setting explains how she would integrate mind wandering as a natural solution to a textual challenge that would allow her to manage the tension and move on. The tensions would be resolved by engaging with a familiar and calming environment that would, in her explanations, equip her with the right kind of break before she can resume the reading task. It seems, in that sense, as if mind wandering is a coping strategy that allows her to complete the task and as such avoid switching task. In contrast, when she feels forced to focus exclusively and permanently on the text, as in the experiment, she is unable to distance herself from it and she is forced to continue without rich opportunities for manipulating the situation. The tension cannot be resolved by use of mental strategies alone, but requires instead, both manipulation of bodily and contextual dimensions. The fixed and controlled setting provides our reader with little control over the cognitive constraints, and it seems detrimental to her capacity to make sense of the text. Voluntarily looking out the window while reading is a good example of an embodied strategy implemented by the agent to modulate the lived time while reading, slowing down, or stopping altogether reading. These embodied strategies allow the reader to vary the deliberate constraints on her thought processes, going from specific forms of mind wandering (like tuning out), to creative thinking, and to goal-directed thought, according to the demands of the situation and the text. Inhibiting that capacity to play with the deliberate constraints on the stream of thoughts leads to negative experiences of reading and in extreme cases to the incapacity to make sense of it.

M’s description of her experience also points to the embodied, situated, and distributed aspects of reading and mind wandering in a more general sense. The field of relevant affordances in reading encompasses not only the text but the whole environment, in which the reader can move around, look out the window, change posture, among many other possible strategies.

### 4.3. Analysis and beyond: An embodied approach involves brain-body-environment systems

The ethnographic and phenomenological analyses are mutually complementary in developing an embodied model of reading. The analyses foreground reading as something a person *does.* Reading is managed by a brain-body-environment system (Järvilehto, 2009) that makes sense and controls sense-making by different embodied pace-making strategies. The reader engages in a reading activity embedded in a specific environment, guided by specific interests and goals, and enacts a wide diversity of strategies in order to give life to the text, to link it to her previous experiences and background, and to turn it into something that “speaks to her.” The picture of reading derived from these analyses is incompatible with the orthodox conception of the reading brain (see LaBerge, 2002). Many densely coupled processes are indeed taking place in reading: affective, bodily, and neurological. And if we are to do justice to the complexity of that activity, a dialogue between the different approaches is needed. Importantly, a constant contact with the phenomena studied is essential to enrich, complement and revise the theories that guide empirical observations.

Crucially, in the point of encounter between the theoretical developments and the empirical observations, we learn how concepts such as mind wandering can be re-interpreted. This re-interpretation is both ontological and epistemological: mind wandering might be better understood as embodied and thus expands the neural boundaries. Further, the concept, we suggest, might have positive effects also on task-management, when viewed over a longer timescale. Specifically, the analyses showed how cases of mind wandering in everyday lives are observable in the fleeting peek through the window, in the raising of the eyebrow, in the change of bodily posture, and even in the act of leaving the room for a glass of water. These phenomena, analyzed with the tools of cognitive ethnography, are paired with the experiences of frustration when the sense-making process fails, or satisfaction when establishing a connection previously overlooked in a text, with the urge to have some “fresh air,” or to move around the room. Such strategies seem functional and valuable for the task at hand. Of course, mind wandering in itself cannot be judged as functional or dysfunctional, it depends on the person, task and situation.

## 5. Discussion: Implications and future directions

The attempt to study mind wandering in reading *in the wild* is not only complex and difficult it is also risky as the results are uncertain. Reading ecologies consist of an array of factors–as we introduced in the section on reading. Every reader is different, every text is different, the medium on which a reader engages spans kindles, iPads, print (books). Texts have different lengths, and different genres afford different pacemaking and rhythmic engagement in the reader. Mind wandering, as a result, emerges within a mix of such constraints and is not just one thing that can be identified unambiguously. The function of mind wandering in reading relies on interpretation of the effects the wandering has for explicit criteria such as imagining, critical thinking, memory, understanding etc. Further, the experiential dimension of mind wandering also has effects on the reader and sometimes the processes can cause emotional tensions such as frustration or even stress. In sum, such video-ethnographic studies reveal the diversity of the effects that mind wandering can have in reading.

There is one more aspect of our proposal that we want to emphasize: the embodied account of mind wandering we proposed here re-introduces the notion of agency, which has been commonly overlooked in the literature. Mind wandering is usually explained in terms of sub personal processes unfolding in specific brain areas, which are on their turn correlated with spontaneous thought processes. This account of mind wandering neglects the contribution of the agent to mind wandering. Instead of thinking of mind wandering as something that *happens to us*, we want to emphasize that many episodes of mind wandering are cases of something *we do*. We often actively and wilfully modulate the conditions that constraint our thought processes, thus enabling the emergence of mind wandering and creative thinking. We do this by modifying the environment, and through a wide array of embodied strategies. This theoretical consideration can be translated into concrete educational plans of action. For instance, one potential outcome could be to train readers’ attention during their own reading processes. If readers are made aware of the potential in scrutinising and eliciting breaks that fuel affective-cognitive ways of thinking, it can be trained. Mind wandering in imaginative reading can be treated as a skill just like the symbolic scanning of letters on a page. We suggest a much more explorative, non-linear and playful approach to imaginative reading where the reader is encouraged to explore in sophisticated ways how their embodied, ruptured engagement has (days) functional effects on the reading experience and outcome.

Finally, let us make explicit a central theoretical claim that constitutes the basis of our proposal: imaginative and creative processes like those unfolding in reading extend beyond the brain of the reader and encompass body and environment. This seems like a trivial consequence of our remarks on embodying reading and mind wandering. But the risk of triviality of this claim is compensated by its enormous theoretical, methodological and practical potentiality. The fact that the imaginative processes associated with specific forms of reading extend to the reader’s body and world implies that they are observable, given the right instruments (the cognitive ethnographic method employed in section “4.1. Case 1: A ruptured reading flow and the imaginative power of breaks” is a good example). Also, if imaginative processes extend to the world, the world around us (including the substrate in which the text is being read) can either enhance or inhibit such imaginative processes. This is still an underexplored area of research, with crucial implications. And, since our surrounding environment is not only material but socio-material, then social norms (like the ones being taught at schools to novice readers) are constitutive of their imaginative processes in ways that have not been explored in relation to embodied reading.

## 6. Conclusion

This article is an invitation to strengthen the dialogue between the different disciplines and fields that contribute to understanding complex phenomena like mind wandering and reading. This invitation is not new, and neither is the idea of integrating neuroscientific accounts into the embodied paradigm. However, despite the growing popularity of embodied cognition, the conceptual and empirical ideal of integrating methods and approaches across disciplines is yet to be achieved. This scarcity of research is the result of conceptual and methodological challenges that necessarily emerge when categories as dissimilar as those of neurocognitive processes, behavioral processes, and experiential reports are integrated into a unitary explanation of a phenomenon (see Martiny et al., 2021). We do not consider this article to be an example of how to fully meet those challenges and completely integrate the different accounts of reading or mind wandering, but we offer some clues about how to carry on that work in future collaborations.

The core of the conceptual work in this article consists of two main developments: (i) A novel understanding of reading as an embodied, messy, and ruptured activity where multiple thought processes co-occur on a continuum and (ii) a proposal of how to enrich neurocognitively centered notion of mind wandering like the one put forth by Christoff and colleagues with an embodied, dynamic model. The first point demanded an embodied turn, which changed the focus from the reading brain in isolation to a brain-body-environment system. Further, this reorientation enables us to consider how different cognitive strategies manifest themselves as embodied, observable and analyzable processes. Secondly, we likewise explored the possibility of conceiving constraints exerted on thought processes not as exclusively mentalistic and neurocognitive entities, but as embodied processes. As such, these constraints encompass not only the neurocognitive level, rather they are also tightly coupled with bodily strategies and environmental conditions, which allow the agent to constrain her thought processes.

In the second part of the article, we showed how our conceptual framework can be applied in empirical research by use of ethnographic observations and phenomenological analyses of experiential reports. We consider both the conceptual and the empirical parts as necessary and mutually dependent in the strengthening of an embodied framework.

## Data availability statement

The raw data supporting the conclusions of this article will be made available by the authors, without undue reservation.

## Ethics statement

Ethical review and approval was not required for the study on human participants in accordance with the local legislation and institutional requirements. The patients/participants provided their written informed consent to participate in this study. Written informed consent was obtained from the individual(s) for the publication of any potentially identifiable images or data included in this article.

## Author contributions

ST conducted a study from which one data set was obtained and the other data set was conducted by ST and JT. Both authors contributed to the theoretical and analytical sections.

## Footnotes

1 In his *Philosophical Investigations* (1953), Wittgenstein invites us to think of the activities we call “games”: “I mean, board-games, card-games, ball-games, athletic games, and so on. What is common to them all?—Don’t say: “they *must* have something in common, or they would not be called games”—but *look and see* whether there is anything in common to all” (§ 66). He concludes that there is not one feature common to all games, but rather “a complicated network of similarities overlapping and criss-crossing” (ibid), which he calls *family resemblances.*

2 One of the main advantages of Seli et al.’s proposal is that it conceives mind wandering as a heterogeneous phenomenon, which demands to be studied in all its nuances and different manifestations. However, when it comes to the application of the notion of mind wandering to specific cases, the proliferation of subtypes makes it problematic to apply it to the case of reading. Since there is an enormous variety of dissimilar mind wandering processes, it would be necessary to assess each one independently, with no clear connection between them. This case reflects the risk that Metzinger (2018), warns against: “in developing a systematic catalog of explananda, we might end up with a very long disjunction (“Mind-wandering is *a* or *b* or *c* or…”) and risk the danger of widespread fallacies of equivocation” (101). The fallacy of equivocation, in informal logic, occurs when there is a misleading use of a term with more than one meaning.

3 Tim Ingold interviewed by Juan Loaiza, June, 2021 for the online ENSO seminar: http://www.ensoseminars.com/presentations/past47/.
